# A Pneumatically Controlled Prosthetic Socket for Transfemoral Amputees

**DOI:** 10.3390/s24010133

**Published:** 2023-12-26

**Authors:** Kang-Ho Lee, Hyun-Seok Heo, Jeongmin Kim, Jang Hyuk Cho, Kyoung Tae Kim, Jeong-Yong Hur, Jang Hwan Kim, Yongkoo Lee

**Affiliations:** 1 Daegu Research Center for Medical Devices, Korea Institute of Machinery and Materials, Daegu 42994, Republic of Korea; jm3900@kimm.re.kr (J.K.); ylee2012@kimm.re.kr (Y.L.); 2 Shinsegae Prosthetic Center, Daegu 41710, Republic of Korea; hhyunsuk0928@naver.com (H.-S.H.); ssg1821@hanmail.net (J.-Y.H.); 3 Department of Rehabilitation Medicine, Keimyung University Dongsan Hospital, Keimyung University School of Medicine, Daegu 42601, Republic of Korea; jangcho@dsmc.or.kr (J.H.C.); zealot42@dsmc.or.kr (K.T.K.); 4 Department of Rehabilitation Technology, Graduate School of Hanseo University, Seosan 31962, Republic of Korea; profpo@naver.com

**Keywords:** transfemoral socket, amputee, pneumatic, air bladder, prosthesis

## Abstract

Amputees typically experience changes in residual limb volume in their daily lives. It causes an uncomfortable fit of the socket by applying high pressure on the sensitive area of the residual limb or by loosening the socket. In this study, we developed a transfemoral prosthetic socket for above-the-knee amputees that ensures a good socket fit by maintaining uniform and constant contact pressure despite volume changes in the residual limb. The socket has two air bladders in the posterior femoral region, and the pneumatic controller is located on the tibia of the prosthesis. The pneumatic system aims to minimize unstable fitting of the socket and improve walking performance by inflating or deflating the air bladder. The developed socket autonomously maintains the air pressure inside the prosthetic socket at a steady-state error of 3 mmHg or less by adjusting the amount of air in the air bladder via closed-loop control. In the clinical trial, amputee participants walked on flat and inclined surfaces. The displacement between the residual limb and socket during the gait cycle was reduced by up to 33.4% after air injection into the socket. The inflatable bladder increased the knee flexion angle on the affected side, resulting in increased stride length and gait velocity. The pneumatic socket provides a stable and comfortable walking experience not only when walking on flat ground but also on slopes.

## 1. Introduction

Amputees use prosthetics to replace their limbs and perform a variety of daily activities [1,2,3]. In particular, lower extremity amputees repeatedly place their body weight on the prosthesis while walking. For such prostheses, the socket surrounding the residual limb must have a stable and comfortable fit to prevent pain in the pressure-sensitive areas and ensure successful rehabilitation [4,5,6]. A good socket fit was achieved by maintaining a uniform pressure distribution around the residual limb. Generally, the actual volume of the residual limb changes in daily life, causing high pressure on the residual limb or looseness of the socket [5,6,7,8,9,10,11]. Therefore, a volume-specific socket that can change over time is required. Recently, studies have been conducted to solve the uncomfortable fitting caused by continuous changes in the residual limb size [5,6]. In [11,12], the socket was tightened or loosened using a dial button, clamps, or a lacing system. These sockets must always be manually manipulated, and their sizes are adjusted based only on the user’s subjective senses without being aware of the exact pressure inside the socket. Studies have been conducted on the measurement of the contact pressure between the residual limb and the socket [13,14,15]. These studies have primarily focused on acquiring pressure too early for use by amputees. Other sockets with air pneumatic functions that adjust the socket fit using an inflatable air bladder have been introduced [16,17,18]. The pneumatic actuator inserts in [16] provide interface pressure mapping and fit improvements. However, actuation control systems are bulky and limit the portable aspect. The pneumatic air suspension system in [17], which is portable, reduces the pressure concentration around the limb; however, it has not been sufficiently validated under changes in the residual limb size. Most sockets exhibit the possibility of being used clinically, but only at the laboratory level [16,18]. Several clinically tested sockets have been reported for amputees. The air socket in [17] was used to investigate the characteristics of the standing posture only. The motor-driven socket [19,20] has been validated through gait but is bulky owing to the motor. Therefore, for commercialization, it is necessary to reduce the apparent volume.

In this study, we propose a pneumatically controlled prosthetic socket to maintain good socket fitting despite volume changes in the residual limb. The proposed transfemoral socket uses air bladders located on both sides of the posterior femoral region. The pneumatic controller was placed on the tibia of the prosthesis to avoid any discomfort, resulting in its proximity to a commercial product. The pneumatic system proposed in this paper weighs only 154 g, which is not much different from the weight of existing sockets. In this study, a clinical test was conducted on transfemoral amputees. The performance of the socket was evaluated by participants walking on flat and inclined surfaces. The displacement between the residual limb and socket was investigated by measuring the knee angle during walking. Changes in stride length and walking velocity were analyzed with and without pneumatic control of the socket. All the experiments were approved by the Keimyoung University Dongsan Hospital Review Board (IRB [DSMC 2021-04-100-017]). The proposed socket technology is expected to be widely used to improve the welfare of amputees.

## 2. Materials and Methods

### 2.1. System Structure and Operation Principle

**Figure 1:**
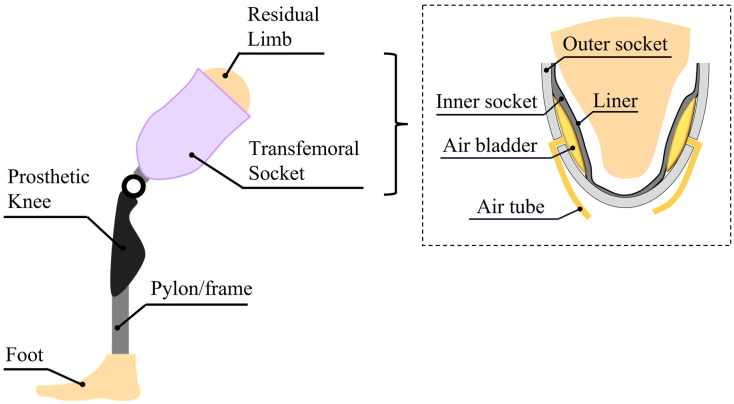
Conceptual diagram of a transfemoral prosthesis with pneumatic system; (inset) the components of a transfemoral socket.

**Figure 2:**
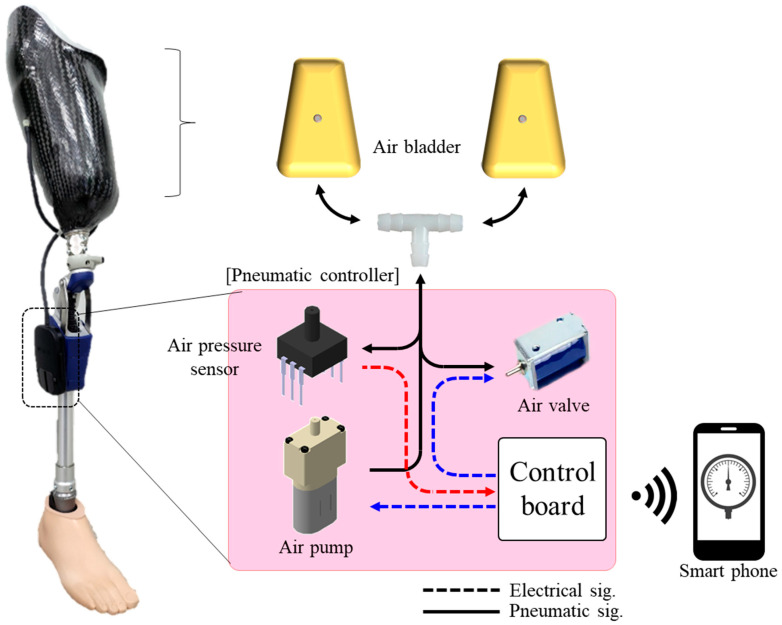
Operation concept of the socket system to pneumatically control the air bladder. The red arrow is the input signal to the control board, and the blue arrow is the signal output from the control board.

Figure 1 shows the transfemoral prosthesis for an above-the-knee amputee. The transfemoral socket comprises a liner composed of silicone rubber, two layers of inner and outer sockets, and air bladders. The liner protects the skin of the residual limb, which is sensitive to irritation and infection. In the case of a transfemoral prosthesis, unlike a transtibial prosthesis, a two-layer socket is generally used. Because the inner socket is in direct contact with the affected thigh area, soft plastic is used to reduce stress on the affected area, groin, and perineum. It also prevents the restriction of muscle movement within the socket. The outer frame socket is made of carbon to ensure durability and support. In the proposed socket, inflatable air bladders are located between the inner and outer sockets. When the air bladder expands, pressure is applied to the limb by lifting the soft inner socket. It provides the effect of applying uniform pressure to a wide area along the curve of the affected area rather than a localized area. Therefore, the air bladder compensates for any loose or tight space between the residual limb and the socket. The air tube is connected to the air bladder and delivers the air flow to the pneumatic controller, as shown in Figure 2.

Figure 2 shows the proposed socket system for pneumatic control of an air bladder. Our limb prosthesis consists of air bladders in the socket, air pump, pneumatic valve, air pressure sensor, and a control board. Air bladders were located on both sides of the back of the femoral region, the interface pressure between the residual limb and the socket was detected in real time, and expansion and contraction were repeated to maintain a constant pressure. The pneumatic controller was located at the side of the tibial pylon to avoid significantly increasing the width of the prosthetic leg. Air is injected by an air pump toward the bladder while being regulated by a pneumatic solenoid valve (solid line). Concurrently, the internal pressure of the air bladder is monitored using a pressure sensor. As the residual limb volume decreases, the air bladder detects a decrease in air pressure and pneumatically inflates the air bladder, resulting in the recovery of the loose fit between the residual limb and socket. An increase in the residual limb size causes an increase in air pressure, which is followed by deflation of the air bladder and relief of excessive pressure. In the socket system, the contact pressure in the socket maintains a specific pressure to return to its original state, despite the change in the volume of the residual limb. In the proposed system, the socket communicates wirelessly with the mobile phone application. The prosthetic user can manually adjust the quantity of air in the socket to reach a comfortable level using a mobile phone. Moreover, the user can monitor the operation status of the pneumatic system, including real-time pressure level and pressure holding time, through a mobile phone. It can also help prevent ischemia by keeping pressure levels within a safe pressure range at all times.

### 2.2. Fabrication of a Pneumatically Controlled Prosthetic Socket

**Figure 3:**
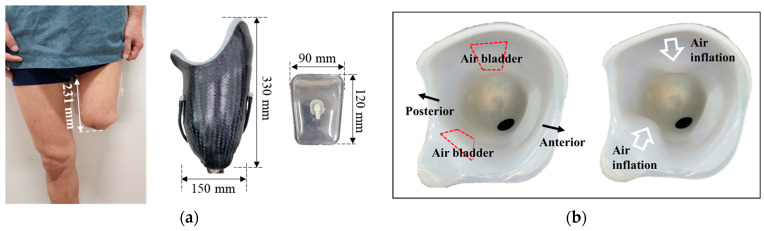

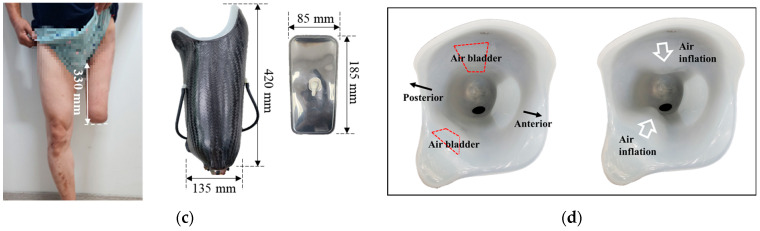
View of femoral limb, fabricated socket, and air bladder of (**a**) Participant #1; (**c**) Participant #2, the inside view of the socket before and after the air bladder’s inflation (**b**) Participant #1; and (**d**) Participant #2.

All the experiments in this study were conducted by two participants with the specifications listed in Table 1. Participant #1 had a shorter limb length and shorter time since surgical amputation than Participant #2. The size of the air bladder must be determined to fabricate a pneumatically controlled prosthetic socket. The size of the air bladder was selected to avoid placing unwanted pressure on the distal end of the stump and ischial ramus. It was located in the posterior femoral area under the ischial ramus, surrounding the residual limb. Therefore, the width and length of the air bladder were determined to approximately 20% of the limb circumference and 50% of the length of the limb, respectively. Figure 3 shows each participant’s socket and the fabricated air bladder. Figure 3b,d show views of the air bladder before and after air injection. As the air bladder inflates, the socket has less space.

### 2.3. Characterization of Air Bladder

**Figure 4:**
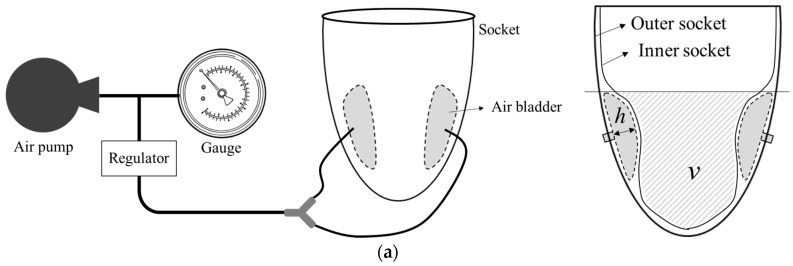

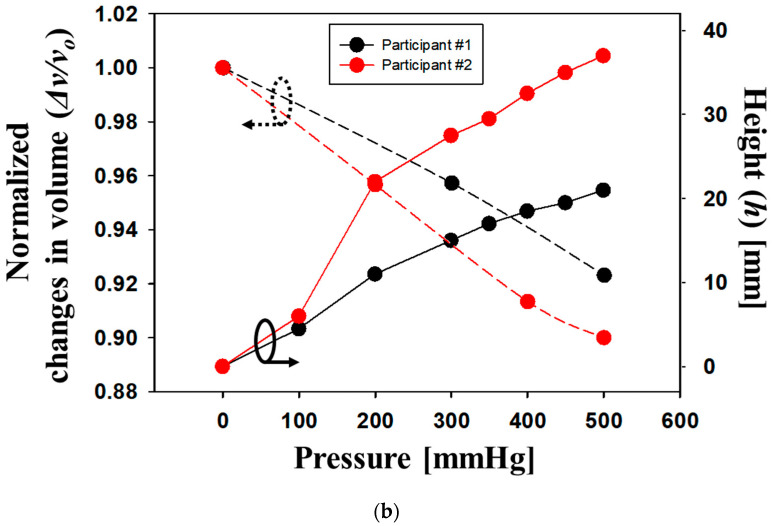
Mechanical properties of the air bladder inserted between inner and outer socket; (**a**) the concept of measurement method; (**b**) normalized changes in volume and height of the air bladder as a function of the applied pressure.

The contact pressure between the socket and residual limb was measured using an air bladder. Based on the measured pressure data, the socket maintains a stable internal pressure by inflating the air bladder at a low pressure or by relieving the air when excessive pressure is applied. Thus, it can compensate for the volume changes in the residual limb. The characteristics of an air bladder, such as size, durable load, expandable volume, and height, determine the performance of an inflatable transfemoral socket. We previously reported the mechanical properties of the air bladder with a maximum height of 50 mm and a maximum weight load of 60 kgf [18]. In this study, the properties of an air bladder located between the inner and outer sockets are investigated. Figure 4 shows the changes in the volume and height of the air bladder inside the socket as a function of the applied pressure. The height of the air bladder was measured directly through the change in vertical length using a gauge tool, and the volume was indirectly measured through the change in displacement of the filled water depending on whether air was injected or not. This experiment was conducted under a no-load condition with only the contact pressure between the inner and outer sockets, and not the inserted limb. We normalized a change in inner space volume, ∆*v*/*v*_0_, which decreased by the inflation of the air bladder. In Figure 4b, the solid lines indicate the bladder height variation in the socket of Participant #1 (black circle) and Participant #2 (red circle). The dotted lines indicate normalized changes in the volume of the inside space. The maximum height of the air bladder inserted between the inner and outer sockets was 37 mm, and the volume inside the socket decreased by 10% at 500 mmHg. This result provides the criteria for compensating for volume changes in the residual limb.

### 2.4. An Alternating Mechanism for Closed- and Open-Loop Control

**Figure 5:**
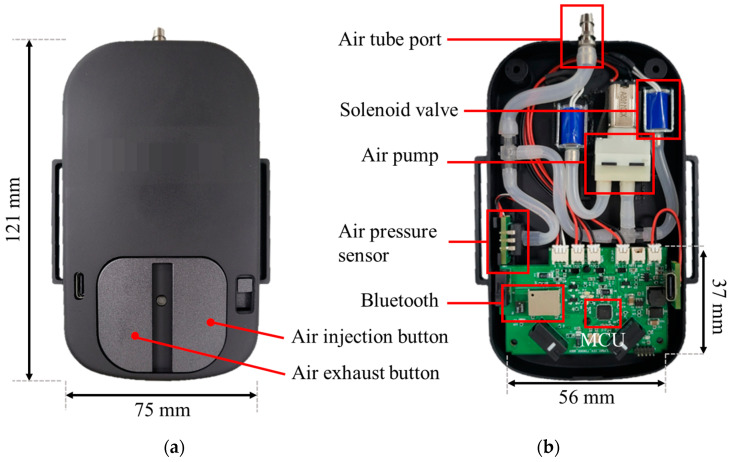
View of the pneumatic controller for socket system; (**a**) top cover; (**b**) inside view of the pneumatic controller.

Figure 5 shows a pneumatic controller with dimensions of 121 mm (L) × 75 mm (W), which is located at the side of the tibial pylon in the prosthesis. The pneumatic controller includes a control-printed circuit board (PCB) with dimensions of 56 mm (L) × 37 mm (W), air pressure sensor, air pump, and a solenoid valve. It used a microcontroller of ATmega328 (Microchip Technology Inc., Chandler, AZ, USA) and was powered by a 3.7 V Li-Po battery consuming 35 mA at normal and 200 mA at full operation of the pump and valve.

**Figure 6:**
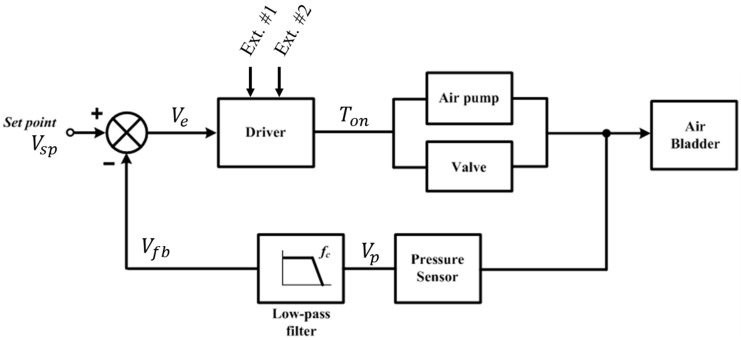
Alternating mechanism for closed- and open-loop control.

We propose an alternating mechanism for closed- and open-loop controls, as shown in Figure 6. The system has a feedback loop to compensate for changes in the volume of the residual limb. The sensed pressure *V_p_* is filtered to *V_fb_* and then compared to the set-point value *V_sp_*, and then the error *V_e_* is converted to on-time *T_on_* for the operation of the air pump and solenoid valve. Here, the driver is based on a comparator model for hysteresis operation introduced in our previous study [18]. The speed of the feedback loop is determined by the cut-off frequency *f_c_* of the low-pass filter (LPF). To adjust the pressure in the air bladder, the air pump and valve were operated as follows:

(1) $$T_{on}=T_{on(Ext.\#1)}+T_{on(Ext.\#2)}+{\alpha \;V}_{e}$$

where *T_on_* denotes the on-time signal for the operation of an air pump or valve. Ext. #1 and Ext. #2 are the External Button #1 for the air-injection function and #2 for the air-exhaustion function. The α is a coefficient that converts voltage signal into time information.

Once the system is powered on, the prosthesis user manually adjusts the air quantity of the air bladder in the socket using an air injection or air exhaust button, as shown in Figure 5. If External Buttons #1 or #2 turn ON, first, the *f_c_* of the LPF is set to be very large, over 100. Thus, *V_fb_* is likely to be *V_p_* because the LPF operates rapidly with a large bandwidth. Second, the set point *V_sp_* is set to be the same as *V_fb_*. Therefore, *V_sp_* is the same as *V_fb_* and *V_p_*, resulting in *V_sp_* being continuously updated to the air pressure signal in real time. Here, the error value (*V_e_*) in the feedback loop was approximately zero. This creates a feedback scheme for the open-loop control. Consequently, manually pushing the external buttons directly regulates the air inside the socket using an open-loop scheme. In the open-loop system, *T_o_* is determined only by the components of Ext. #1 and Ext. #2, as follows:

(2) $$T_{on}=T_{on(Ext.\#1)}+T_{on(Ext.\#2)}$$

If both the External Buttons #1 and #2 are OFF, the cut-off frequency of the LPF is first set to a specific value of 0.001 or less. This indicates that the feedback loop in the proposed system is extremely slow. This is because the volume of the residual limb to be compensated by our system changes over long periods in daily life. Second, *V_sp_* is fixed at a specific value of *V_fb_* just before all external buttons are turned OFF, whereas *V_fb_* is continuously updated via the LPF. Therefore, *V_e_* has a finite value other than zero, and as a result, the feedback loop is completed in the form of a closed-loop control. In a closed-loop system, *T_o_* is determined only by the error signal of the feedback loop as follows:

(3) $$T_{on}={\alpha \;v}_{e}$$

In our socket system, the alternating mechanism for the closed- and open-loop control was determined by the ON/OFF states of the external buttons.

(4) $$\left\{\;\begin{array}{c} \mathrm{Open}\;\mathrm{loop}\;\mathrm{control}\;:\;V_{SP\;}=V_{fb\;}≅V_{p\;}\left(large\;f_{c\;}\right),\;\mathrm{when}\;\mathrm{Ext}.\;\mathrm{buttons}\;\mathrm{ON}\;\\ \mathrm{Closed}\;\mathrm{loop}\;\mathrm{control}\;:\;V_{SP\;}\neq V_{fb\;}\neq V_{p\;}\left(small\;f_{c\;}\right),\mathrm{when}\;\mathrm{Ext}.\;\mathrm{buttons}\;\mathrm{OFF}\; \end{array}\right.$$

## 3. Results and Discussion

### 3.1. Verification of a Closed-Loop Control

**Figure 7:**
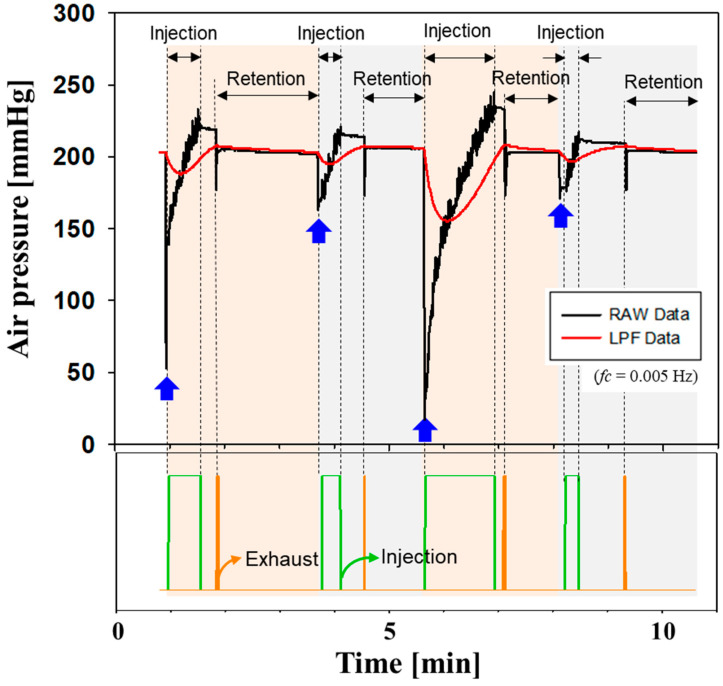
Verification of a closed-loop control in pneumatically controlled socket.

The proposed socket system autonomously maintains the air pressure of the prosthetic socket using a closed-loop control. Figure 7 shows the operation of the pneumatic socket using the closed-loop control. The steady-state error *ε_ss_* of air pressure was evaluated by causing repulsive leakage from the air bladder. The blue arrows indicate the occurrence of air leakage followed by an abrupt decrease in air pressure. The set-point pressure and cut-off frequency in the feedback loop were 203 mmHg and 0.005 Hz, respectively. Through a closed-loop operation, air is injected toward the bladder in the socket for the green signal duration. Once the LPF data reach a specific level of pressure, air starts to be exhausted for the orange duration; therefore, the air pressure is settled at the set-point level. The proposed socket system successfully maintained a predefined set-point value of 203 mmHg with a maximum steady-state error of 3 mmHg despite various changes in volume. Our socket is expected to compensate for volume changes in the residual limb.

### 3.2. Performance of Pneumatically Controlled Prosthetic Socket

**Figure 8:**
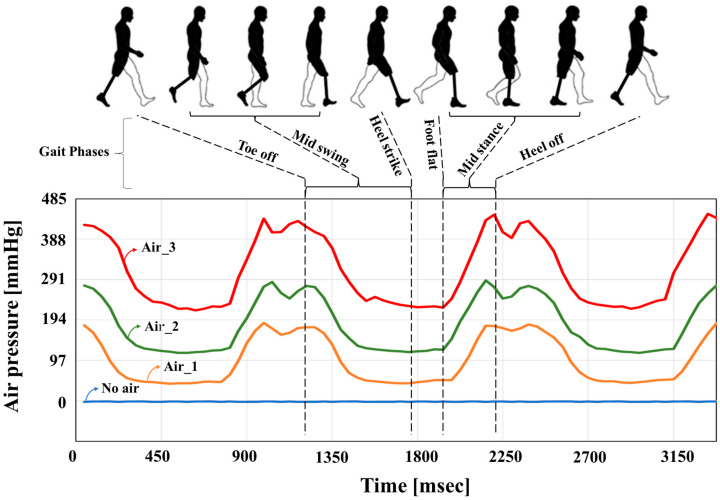
Change in pressure inside the air bladder when walking under different air quantities in the socket. In the standing position, the pressures in Air_1, Air_2, and Air_3 were 70, 135, and 225 mmHg, respectively.

The performance of the proposed socket was investigated using the gait patterns of participants with transfemoral amputees (Table 1). The participants walked on flat ground at a distance of 6.5 m. Figure 8 shows the change in pressure inside the air bladder when Participant #1 walked under different air quantities (No air, Air_1 < Air_2 < Air_3) in the socket. In the standing position, the pressures in Air_1, Air_2, and Air_3 were 70, 135, and 225 mmHg, respectively. The internal pressure of the air bladder was 0 mmHg when air was not injected. Here, we clearly acquired the gait pattern (phases: Toe off—Mid swing—Heel strike—Foot flat—Mid stance—Heel off) of the walker using the pressure dataset of the air bladder, as shown in Figure 8. As the intensity of the air in the socket increased, the offset level of the air pressure increased. Bumps in the Heel off and Toe off phases were clearly observed at a higher air injection intensity.

**Figure 9:**
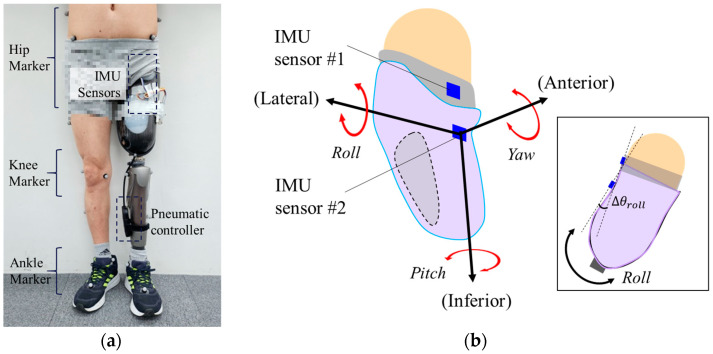
(**a**) Experimental set-up and (**b**) method for evaluating displacement between the residual limb and socket during walking. The difference in rolling angles $∆\theta_{Roll}$ between IMU Sensor #1 and IMU Sensor #2 was measured.

We evaluated the displacement between the residual limb and socket during the gait cycle, which represents the socket fit. Ideally, the movement of the residual limb must be identical to that of the socket, without any unwanted motion. However, if the volume of the residual limb changes, and the socket fit is poor, unwanted repetitive displacement occurs inside the socket, causing pain. As shown in Figure 9, two inertial measurement units (IMUs) (BNO080, CEVA Inc., Mountain View, CA, USA) were attached to the liner and socket of the prosthesis. IMU #1 on the liner and IMU #2 on the socket provided data related to the movements of the residual limb and socket, respectively. In particular, when the leg swung along a straight line while walking, the displacement could be identified by the rolling angle relative to the lateral axis, as shown in Figure 9b. Therefore, we are interested in the difference in rolling angles $∆\theta_{Roll}$ between IMU Sensor #1 and IMU Sensor #2 in the inset of Figure 9b.

**Figure 10:**
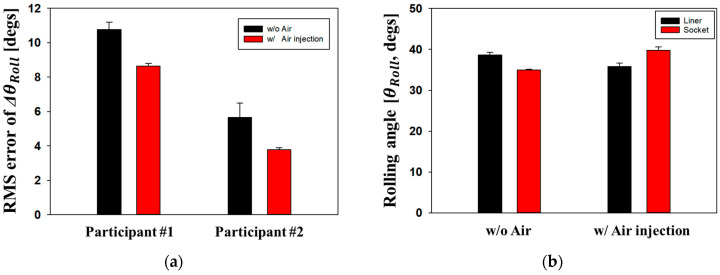
(**a**) Root-mean-square (RMS) error of $∆\theta_{Roll}$ during Mid-stance walking phase; (**b**) the rolling angle ($\theta_{Roll}$) from IMU Sensor #1 when the participant walks on flat ground.

Figure 10a shows the root-mean-square (RMS) error of $∆\theta_{Roll}$ during the Mid-stance walking phase when the participants walked on flat ground. The Mid-stance walking phase is advantageous for observing the displacement between the residual limb and socket because most of the body weight was placed only on the prosthesis leg. If the socket fit is poor, the residual limbs may roll further than the socket. The RMS errors of $∆\theta_{Roll}$ for Participants #1 and #2 decreased to 19.8% and 33.4% following air injection of 225 mmHg and 180 mmHg, respectively. The air-inflatable socket significantly decreased the variation in the residual limb inside the socket, thereby providing comfort and stable walking. Here, RMS errors of $∆\theta_{Roll}$ for Participant #2 were relatively smaller than those of Participant #1. This is because Participant #2′s longer amputation limb originally increased the contact area between the residual limb and the socket, resulting in much less vibration in the socket. Figure 10b shows the rolling angle ($\theta_{Roll}$) of the residual limb measured by IMU Sensor #1 during Participant #1′s walking. The rolling ratio, δ, during the gait cycle is defined as follows:

(5) $$Rolling\;raio\left(\delta \right)during\;gait\;cycle=\frac{Rolling\;angle\;of\;Liner}{Rolling\;angle\;of\;Socket}$$

Large δ indicates that the displacement of the residual limb is unnecessarily large relative to the movement of the socket, resulting in rapid limb fatigue. In a normal socket without air, the walker’s residual limb has a greater rolling angle compared to the socket (δ_1_ = 1.08). On the other hand, when air is injected into the socket, the rolling angle of the residual limb is reduced (δ_2_ = 0.88). It is understood that walking motion has improved by approximately 18.6% ((δ_1_ − δ_2_) × 100/δ_1_) owing to the inflatable socket.

**Figure 11:**
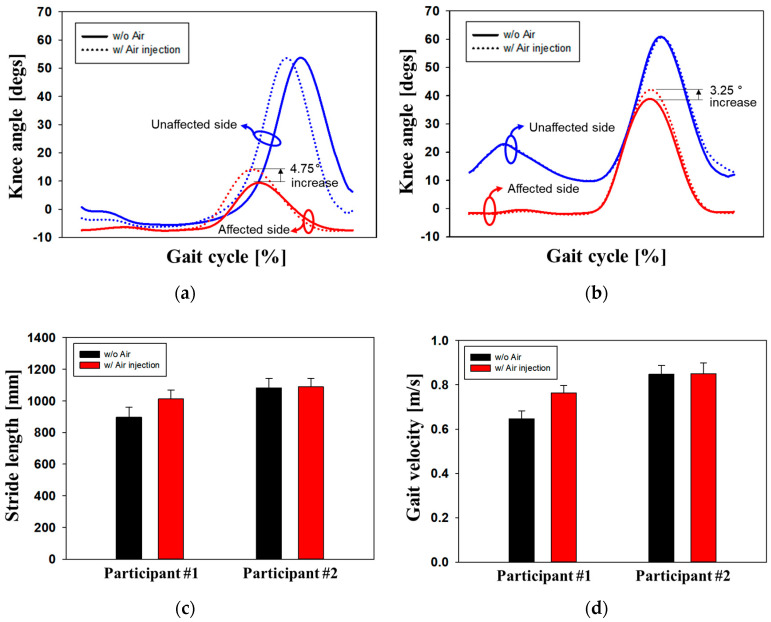
Knee angles as gait cycle of (**a**) Participant #1 and (**b**) Participant #2; (**c**) stride length; and (**d**) gait velocity, which are measured using a motion analysis system.

In this study, the gait characteristics of walkers with an inflatable socket were evaluated using optical motion analysis equipment (Vicon Motion System Ltd., Oxford, UK) [21,22]. Several markers were attached to the hip, knee, ankle, and foot, as shown in Figure 9a. Figure 11a,b shows the knee angles of Participants #1 and #2 during the gait cycle. The unaffected side of the normal limb is represented by blue lines while the affected side of the prosthetic leg is represented by red lines. Generally, the knee flexion angle on the unaffected side is larger than that on the affected side because the walker generates a driving force using the unaffected side. For Participant #1, as shown in Figure 11a, when using a socket with an air pressure of 225 mmHg, the knee angle on the affected side increased by 4.75° compared with when using the socket without air. For Participant #2, as shown in Figure 11b, the knee angle on the affected side increased by 3.25°when using an air-injected socket at 180 mmHg. The similarity between the affected side and the unaffected sides improved by approximately 8.87% and 5.34% for Participants #1 and #2, respectively, owing to the air injection socket. Both participants maintained the same knee angle on their unaffected side with or without air. We evaluated the stride length and gait velocity with and without air inside the socket, as shown in Figure 11c,d, respectively. For Participant #1, stride length and gait velocity increased by 12.9% and 16.9%, respectively, whereas Participant #2 showed no improvement. Participant #2 had undergone surgical amputation a long time ago and, as a result, became accustomed to the existing prosthetic socket. Participant #2 requires sufficient walking training with the newly proposed socket.

**Figure 12:**
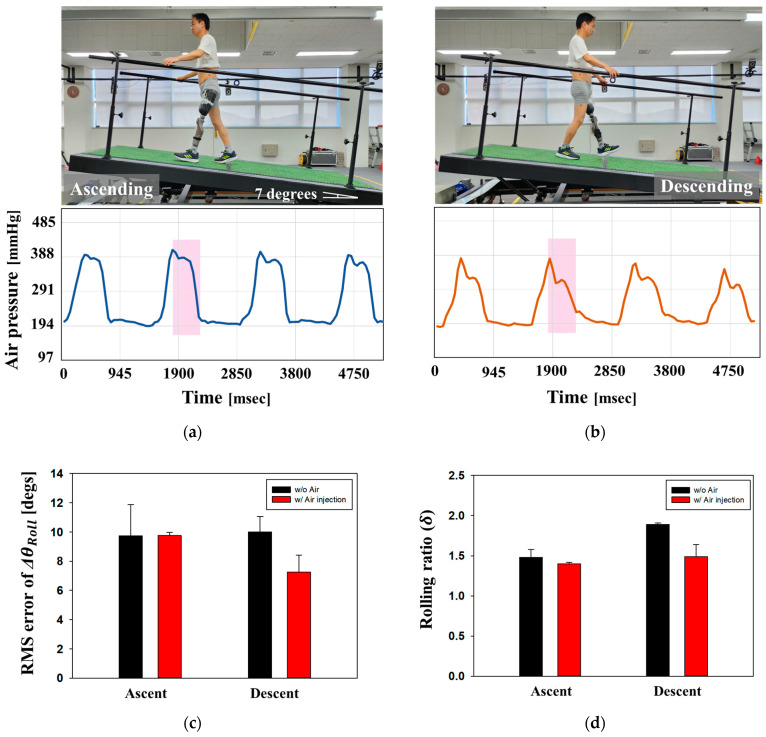
Evaluation of gait on slope with an incline of 7°. Internal pressure of the air bladder in socket when walker (**a**) ascends; (**b**) descends; (**c**) the RMS error of $∆\theta_{Roll}$; and (**d**) the rolling ratio δ in ascending and descending walking.

As shown in Figure 12, the gait of Participant #1 was evaluated using a slope with an inclination of 7°. Figure 12a,b shows the internal pressure of the air bladder in the socket when the walker ascended and descended on the slope, respectively. Different gait phase patterns were observed during the Heel off—Toe off—Mid swing phases (pink block). This is because the prosthetic leg was lifted off the ground to quickly shift the body’s center of gravity when descending. The air pressure patterns enabled us to determine whether the walking direction was upward or downward. Figure 12c shows the RMS error of $∆\theta_{Roll}$ when the participant ascends and descends on the slope. When ascending, the error of the $∆\theta_{Roll}$ is similar, regardless of whether the socket is with or without air. However, in the case of the descent of the slope, the RMS error of decreased to 27.3% when using the air-injected socket. Figure 12d shows the rolling ratio, δ, in ascending and desending walking. When descending, the rolling angle of the residual limb in the air-injected socket was reduced by 21.2% compared to a normal socket without air, resulting in reduced fatigue in terms of walking efficiency.

## 4. Conclusions

In this study, we developed a pneumatically controlled prosthetic socket that maintains good socket fitting despite volume changes in the residual limb during daily life. The proposed socket uses an inflatable air bladder to detect the contact pressure between the residual limb and the socket. The pneumatic system aims to minimize unstable fitting of the socket and improve walking performance by inflating or deflating the air bladder. The fabricated transfemoral socket, which is close to a commercial product, contains air bladders on both sides of the posterior femoral region, and the pneumatic controller is located on the tibia of the prosthesis to avoid any discomfort. In this study, a clinical test was conducted on two participants with amputees over the knee. The performance of the proposed socket was verified using walking patterns on both flat and inclined surfaces. The displacement between the residual limb and socket during the gait cycle was evaluated through the difference in the rolling angles $∆\theta_{Roll}$ of the liner and socket. The RMS errors of $∆\theta_{Roll}$ for Participants #1 and #2 significantly decreased to 19.8% and 33.4% after air injection. The inflatable bladder also reduced the rolling angle of the residual limb, resulting in reduced limb fatigue. The knee flexion angles on the affected side of Participants #1 and #2 increased by 4.75°and 3.25°, respectively, thereby improving the similarity to the unaffected side. In particular, for Participant #1, stride length and gait velocity increased by 12.9% and 16.9% when using the socket with air. When descending a slope with an incline of 7°, the air-injected socket resulted in a 27.3% reduction in RMS error of $∆\theta_{Roll}$ and a 21.2% reduction in rolling angle. Consequently, the pneumatic socket provided a stable and comfortable walking experience. In the proposed socket, the gait patterns can be transmitted to the walker’s mobile phone, providing pressure information inside the socket. These gait patterns can be utilized to investigate the gait balance in walkers, displacement and fatigue of the residual limb, and socket misalignment. In the future, our study should be verified with more participants. To standardize socket fittings, we will develop quantitative standards to judge the quality of socket fittings.

## Figures and Tables

**Table 1:** Specifications of participant’s femoral limb.

Participant	Gender	Age (y)	Circumference of Limb (cm)	Length of Limb (cm)	Time Since Amputation (y)
#1	M	57	42	23.1	8
#2	M	67	40	33	22

## Data Availability

The data presented in this study are available on request from the corresponding author.
